# Supplementary figures and images for: Recognition of Conus species using a combined approach of supervised learning and deep learning-based feature extraction
Source: PLoS One. 2024 Dec 9;19(12):e0313329. doi: 10.1371/journal.pone.0313329 (PMC11627371; doi:10.1371/journal.pone.0313329)

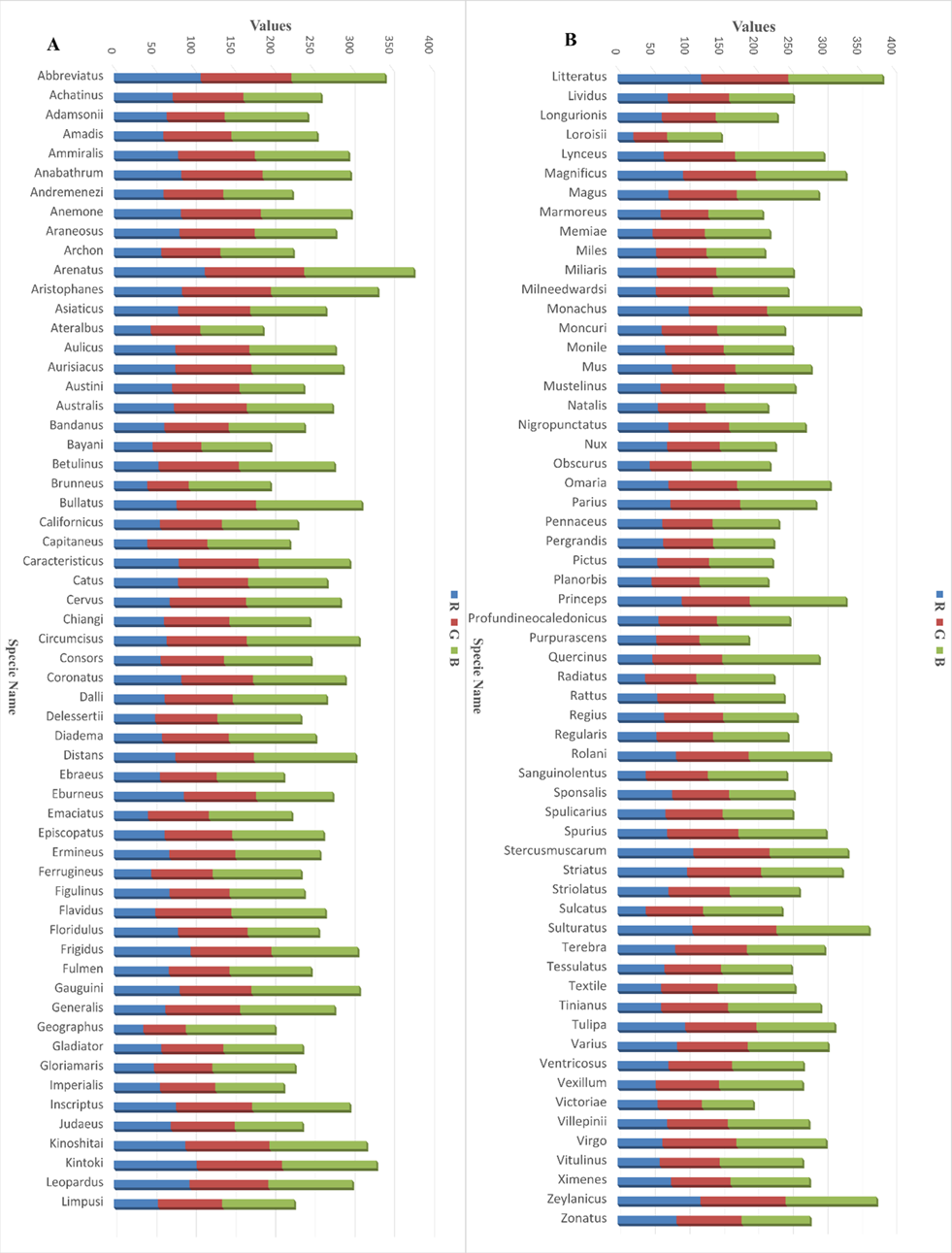

Supplement: S1 Fig — Average predicted values were 70.23 for R, 88.12 for average G, and 107.98 for B. A) First 59 species (X-axis) with their respective RGB values (Y-axis). B) Last 60 species (X-axis) with their respective RGB values (Y-axis). (TIF) [file pone.0313329.s001.tif]
