# Supplementary material for: Recognition of Conus species using a combined approach of supervised learning and deep learning-based feature extraction
Source: PLoS One. 2024 Dec 9;19(12):e0313329. doi: 10.1371/journal.pone.0313329 (PMC11627371; doi:10.1371/journal.pone.0313329)
Supplement: S1 Table — Highlighted five rows indicate wrong predictions. (DOCX) [file pone.0313329.s002.docx]

Table S1: Prediction results of species. Highlighted five rows showing incorrect predictions against species images.

| Image | Predicted Species | Image | Predicted Species |
| --- | --- | --- | --- |
| Conus pergrandis_203.jpg | *Conus pergrandis* | Conus anemone_202.jpg | *Conus anemone* |
| Conus terebra_419.jpg | *Conus terebra* | Conus ebraeus_113.jpg | *Conus ebraeus* |
| Conus aulicus_128.jpg | *Conus aulicus* | Conus regius_197.jpg | *Conus regius* |
| Conus vexillum_41.jpg | *Conus vexillum* | Conus dalli_419.jpg | *Conus dalli* |
| Conus californicus_349.jpg | *Conus californicus* | Conus varius_112.jpg | *Conus varius* |
| Conus bullatus_284.jpg | *Conus bullatus* | Conus catus_2.jpg | *Conus catus* |
| Conus gloriamaris_174.jpg | *Conus gloriamaris* | Conus cervus_415.jpg | *Conus cervus* |
| Conus spulicarius_107.jpg | *Conus spulicarius* | Conus diadema_309.jpg | *Conus diadema* |
| Conus Rattus_33.jpg | *Conus Rattus* | Conus imperialis_373.jpg | *Conus imperialis* |
| Conus limpusi_4.jpg | *Conus limpusi* | Conus stercusmuscarum_44.jpg | *Conus stercusmuscarum* |
| Conus aurisiacus_252.jpg | *Conus aurisiacus* | Conus capitaneus_463.jpg | *Conus capitaneus* |
| Conus archon_409.jpg | *Conus archon* | Conus andremenezi_351.jpg | *Conus andremenezi* |
| Conus litteratus_433.jpg | *Conus litteratus* | Conus geographus_45.jpg | *Conus geographus* |
| Conus zeylanicus_145.jpg | *Conus zeylanicus* | Conus judaeus_412.jpg | *Conus judaeus* |
| Conus pictus_373.jpg | *Conus pictus* | Conus ateralbus_427.jpg | *Conus ateralbus* |
| Conus coronatus_410.jpg | *Conus coronatus* | Conus obscurus_389.jpg | *Conus obscurus* |
| Conus ventricosus_453.jpg | *Conus ventricosus* | Conus mustelinus_76.jpg | *Conus mustelinus* |
| Conus zonatus_136.jpg | *Conus zonatus* | Conus planorbis_277.jpg | *Conus planorbis* |
| Conus arenatus_76.jpg | *Conus arenatus* | Conus kinoshitai_408.jpg | *Conus kinoshitai* |
| Conus tulipa_31.jpg | *Conus tulipa* | Conus kintoki_1.jpg | *Conus kintoki* |
| Conus achatinus_228.jpg | *Conus achatinus* | Conus milneedwardsi_25.jpg | *Conus milneedwardsi* |
| Conus inscriptus_421.jpg | *Conus inscriptus* | Conus gauguini_203.jpg | *Conus gauguini* |
| Conus parius_43.jpg | *Conus parius* | Conus australis_326.jpg | *Conus australis* |
| Conus lividus_228.jpg | *Conus lividus* | Conus leopardus_329.jpg | *Conus leopardus* |
| Conus mus_381.jpg | *Conus mus* | Conus memiae_145.jpg | *Conus memiae* |
| Conus betulinus_89.jpg | *Conus betulinus* | Conus flavidus_219.jpg | *Conus betulinus* |
| Conus abbreviatus_437.jpg | *Conus abbreviatus* | Conus striatus_294.jpg | *Conus striatus* |
| Conus monile_471.jpg | *Conus kintoki* | Conus eburneus_219.jpg | *Conus eburneus* |
| Conus generalis_91.jpg | *Conus generalis* | Conus striolatus_120.jpg | *Conus striolatus* |
| Conus sponsalis_430.jpg | *Conus sponsalis* | Conus ferrugineus_468.jpg | *Conus ferrugineus* |
| Conus bayani_416.jpg | *Conus bayani* | Conus purpurascens_140.jpg | *Conus purpurascens* |
| Conus miles_279.jpg | *Conus miles* | Profundiconus neocaledonicus_43.jpg | *Profundiconus neocaledonicus* |
| Conus aristophanes_483.jpg | *Conus aristophanes* | Conus sanguinolentus_218.jpg | *Conus sanguinolentus* |
| Conus loroisii_55.jpg | *Conus loroisii* | Conus quercinus_211.jpg | *Conus quercinus* |
| Conus virgo_33.jpg | *Conus monachus* | Conus asiaticus_476.jpg | *Conus asiaticus* |
| Conus fulmen_118.jpg | *Conus fulmen* | Conus monachus_74.jpg | *Conus monachus* |
| Conus frigidus_426.jpg | *Conus frigidus* | Conus lynceus_20.jpg | *Conus lynceus* |
| Conus caracteristicus_154.jpg | *Conus caracteristicus* | Conus magus_303.jpg | *Conus magus* |
| Conus ximenes_215.jpg | *Conus ximenes* | Conus bandanus_89.jpg | *Conus bandanus* |
| Conus tinianus_205.jpg | *Conus catus* | Conus brunneus_229.jpg | *Conus brunneus* |
| Conus villepinii_245.jpg | *Conus villepinii* | Conus textile_475.jpg | *Conus textile* |
| Conus vitulinus_76.jpg | *Conus regularis* | Conus ammiralis_241.jpg | *Conus ammiralis* |
| Conus distans_284.jpg | *Conus distans* | Conus consors_213.jpg | *Conus consors* |
| Conus victoriae_3.jpg | *Conus victoriae* | Conus anabathrum_297.jpg | *Conus anabathrum* |
| Conus episcopatus_88.jpg | *Conus episcopatus* | Conus pennaceus_428.jpg | *Conus pennaceus* |
| Conus sulturatus_156.jpg | *Conus sulturatus* | Conus austini_13.jpg | *Conus austini* |
| Conus miliaris_78.jpg | *Conus miliaris* | Conus floridulus_318.jpg | *Conus floridulus* |
| Conus ermineus_395.jpg | *Conus ermineus* | Conus longurionis_17.jpg | *Conus longurionis* |
| Conus radiatus_135.jpg | *Conus radiatus* | Conus araneosus_493.jpg | *Conus araneosus* |
| Conus amadis_351.jpg | *Conus amadis* | Conus spurius_246.jpg | *Conus spurius* |
| Conus omaria_413.jpg | *Conus omaria* | Conus princeps_152.jpg | *Conus princeps* |
| Conus circumcisus_196.jpg | *Conus circumcisus* | Conus chiangi_352.jpg | *Conus chiangi* |
| Conus delessertii_173.jpg | *Conus delessertii* | Conus marmoreus_317.jpg | *Conus marmoreus* |
| Conus rolani_120.jpg | *Conus rolani* | Conus nux_414.jpg | *Conus nux* |
| Conus figulinus_96.jpg | *Conus figulinus* | Conus magnificus_376.jpg | *Conus magnificus* |
| Conus emaciatus_356.jpg | *Conus emaciatus* | Conus moncuri_305.jpg | *Conus moncuri* |
| Conus natalis_57.jpg | *Conus natalis* | Conus tessulatus_259.jpg | *Conus tessulatus* |
| Conus regularis_297.jpg | *Conus regularis* | Conus nigropunctatus_264.jpg | *Conus nigropunctatus* |
| Conus gladiator_296.jpg | *Conus gladiator* |  |  |
