# Supplementary material for: Recognition of Conus species using a combined approach of supervised learning and deep learning-based feature extraction
Source: PLoS One. 2024 Dec 9;19(12):e0313329. doi: 10.1371/journal.pone.0313329 (PMC11627371; doi:10.1371/journal.pone.0313329)
Supplement: Attachment — Submitted filename: Response to Reviewers.docx [file pone.0313329.s004.docx]

Editor

PLOS ONE

Dear Editor,

On behalf of all coauthors, I would like to thank you and both reviewers for the valuable feedback to enhance the quality of our manuscript entitled “**Recognition of *Conus* Species Using a Combined Approach of Supervised Learning and Deep Learning-Based Feature Extraction”**.

**1. Proofread the entire manuscript.**

We have thoroughly reviewed the manuscript for any grammatical errors and sentence structure anomalies. All sections have been carefully proofread and issues have been resolved. We believe that the manuscript now meets the standards expected for publication in terms of readability.

**2. Compare current approach with previous approaches.**

We have carefully addressed the comments raised by worthy reviewers. We appreciate the opportunity to revise the manuscript and resubmit it for reconsideration.

Thank you for your valuable time.

Sincerely,

Sajid Rashid

[sajid@qau.edu.pk](mailto:sajid@qau.edu.pk)
